# A digital recipe for enhancing clinical reasoning: the role of e-learning by concordance (E-LbC): a quasi-experimental study

**DOI:** 10.1186/s12909-025-08005-w

**Published:** 2025-10-02

**Authors:** Hadeel Aboueisha, Enjy Abouzeid, Moataz A. Sallam, Wagdy Talaat

**Affiliations:** 1 Medical Education Department, Suez Canal University Hospitals, Ismailia, Egypt; 2 School of Medicine, Ulster University, Derry~Londonderry, UK; 3 Ophthalmology Department, Suez Canal University – Faculty of Medicine, Ismailia, 41522 Egypt

**Keywords:** Electronic learning by concordance (e-LbC), Script concordance test (SCT), And clinical reasoning (CR)

## Abstract

**Background:**

Clinical reasoning (CR) is a critical competency in medical education, essential for effective decision-making in clinical practice. This study aimed to enhance CR skills among undergraduate medical students by comparing two instructional strategies: the E-learning by Concordance (e-LbC) approach and an interactive lecture-based method.

**Methods:**

A quasi-experimental comparative study was conducted at the Faculty of Medicine, Suez Canal University, Egypt, during the 2021–2022 academic year. The study involved 60 fifth-year medical students through comprehensive sampling and was implemented over one academic term. It consisted of three phases. In the first phase, an online Script Concordance Test (SCT) was used via the Wooclap platform to assess students’ baseline CR skills. The second phase included the educational intervention, in which the e-LbC method was used to teach the topic of painless vision loss, while the interactive lecture method was used for painful vision loss. In the final phase, a researcher-developed questionnaire assessed students’ perceptions regarding the impact of each instructional method on CR development, difficulty level, and satisfaction. The questionnaire’s validity was established by medical education experts, and reliability was confirmed using Cronbach’s alpha.

**Results:**

Statistical analysis using paired t-tests revealed no significant difference in the pre-SCT scores between groups. However, post-SCT scores showed a statistically significant improvement in both groups, with the e-LbC, painless vision loss theme, demonstrating a greater effect size (Cohen’s d) and overall higher performance (*p* < 0.001). Additionally, 62% of students expressed satisfaction with the e-LbC method.

**Conclusion:**

the e-LbC approach positively influenced students’ clinical reasoning skills and engagement. Its integration with real-time assessment tools like Wooclap, combined with its cost-effectiveness, flexibility, and user-friendliness, positions it as a valuable tool for enhancing medical education in diverse learning environments.

**Supplementary Information:**

The online version contains supplementary material available at 10.1186/s12909-025-08005-w.

## Introduction

Clinical reasoning (CR) is widely recognized as a foundational competence in both health professional education and clinical practice [1]. As such, structured clinical reasoning curricula are essential in undergraduate medical education. However, teaching this skill poses significant pedagogical challenges, particularly among undergraduate learners.

Clinical reasoning can be defined as a skill, process, or outcome by which clinicians observe, collect, and interpret clinical data to diagnose and treat patients [2]. Research shows that medical expertise is less about memory or problem-solving alone and more about how knowledge is structured and applied through individualized scripts developed via learning and experience [3]. Expert clinicians often make rapid and accurate diagnoses by drawing on these internalized cognitive frameworks known as illness scripts [4].

The theoretical underpinnings of this study draw on script theory and Croskerry’s dual-process theory, both of which illuminate how clinicians think and make decisions in complex clinical contexts. Script theory suggests that clinicians retrieve relevant mental models, illness scripts, based on prior knowledge and experience [2]. Croskerry’s dual-process theory differentiates between two modes of reasoning: intuitive, experience-based (Type 1), and analytical, deliberate (Type 2). Both are used in tandem depending on the clinical scenario [5, 6].

Learning by Concordance (LbC) builds directly on these theoretical foundations. It offers a structured and authentic method to engage learners in clinical reasoning under uncertainty, promoting the development of robust illness scripts and supporting both Type 1 and Type 2 reasoning processes [5, 7]. LbC simulates real-life decision-making by presenting learners with contextualized clinical scenarios, asking them to make judgments, and comparing their responses with those of expert panels.

LbC follows the model of a cognitive apprenticeship, allowing learners to observe expert thinking and progressively gain independence as their reasoning skills evolve [8]. Unlike traditional instructional models that emphasize knowledge acquisition followed by application, LbC integrates both processes within a single, interactive experience [9]. Its versatility allows it to be adapted to various domains, including clinical decision-making, ethics, and professionalism [10]. With advances in educational technology, LbC can now be delivered online (e-LbC), making it accessible, scalable, and convenient for a broad range of learners [11].

Prior studies have demonstrated the effectiveness of LbC in medical fields such as ECG interpretation and oral pathology. For example, Charton and colleagues found that LbC fostered deeper reflection among future general practitioners by prompting learners to compare their decisions with expert justifications [10]. Another study highlighted how LbC supported clinical reasoning development in dental education through a low-cost and scalable platform [12].

Roche et al. (2025) conducted a scoping review of the Learning-by-Concordance (LbC) approach in health professions education and found that, although interest in LbC is growing, the literature remains limited and heterogeneous, reflecting its innovative and emerging nature [13]. The approach has been applied across various learner types, disciplines, and contexts [10, 11, 13, 14] Most studies used cohort-based follow-up designs to investigate learner engagement and implementation strategies [10, 11, 13, 14].

Learners consistently reported positive experiences, including enhanced engagement, interactivity, and support for structuring clinical reasoning [5, 7, 11, 15–18]. However, these study designs do not allow determination of the added value of LbC compared with other educational approaches such as problem-based learning or case-based discussions [13]. The asynchronous digital format was especially appreciated for its flexibility, enabling learners to progress at their own pace [12, 19, 20].

Moreover, existing research has not sufficiently explored LbC in ophthalmology, a specialty that relies heavily on visual pattern recognition and diagnostic accuracy. Also, comparative evaluations between LbC and traditional instructional methods, such as interactive lectures, remain scarce in this domain. This gap limits our understanding of LbC’s broader applicability across medical specialties.

Accordingly, this study aims to evaluate the effectiveness of electronic Learning by Concordance (e-LbC) in enhancing clinical reasoning skills among undergraduate medical students during an ophthalmology clerkship. Specifically, it compares the effect of e-LbC instruction of painless vision loss theme with that of traditional interactive lectures of painful vision loss theme. The study raises the following question and hypothesis:

### Research question

Does the e-LbC instructional method improve clinical reasoning in undergraduate medical students more effectively than traditional interactive lectures?

### Hypothesis

Students exposed to e-LbC will demonstrate greater improvement in clinical reasoning, as measured by SCT scores, compared to those receiving traditional lecture-based instruction.

## Materials and methods

### Study design & participants

This quasi-experimental study (pre-test/post-test design) was conducted during the 2021–2022 academic year at the Faculty of Medicine, Suez Canal University. The Ophthalmology clerkship spanned two academic terms, each comprising three consecutive rounds. Each round lasted six weeks and included 20 fifth-year medical students, resulting in a total of 60 students per term. A comprehensive sample of 60 students enrolled in the second-term Ophthalmology clerkship was designated as the study group. These students participated in the intervention and were assessed for clinical reasoning using the Script Concordance Test (SCT).

An independent pilot sample of 30 students was selected from the 60 students enrolled in the first-term clerkship. This sample was used solely for piloting and validating the SCT instrument prior to its use in the main study. The pilot sample size followed the general rule of thumb for test validation—10% to 20% of the main study sample, or a minimum of 30–50 participants [10]—to ensure adequate content validity and clarity of the test items.

The Ophthalmology branch was selected from among the major branches in the fifth year of medical education as its staff were well-trained on learning by concordance. Additionally, they depended on SCT in their formative assessment. All participants in the second-term group (study group) were exposed to two modes of instruction. They experienced painless vision loss through E-LBC and painful vision loss through an interactive lecture. This design was adopted to ensure that all students had a similar learning experience while avoiding any ethical concerns related to depriving one group of an experience. Additionally, it eliminated the need for a crossover design.

To ensure that any observed effect could be attributed to the instructional method rather than external factors, we adopted several steps:

1. Baseline Assessment: A pre-test was administered to all students to establish their baseline knowledge and minimize the influence of prior information as a confounding variable.
2. Standardized Assessment Tool: All participants were evaluated using the same Script Concordance Test (SCT) to control for potential bias arising from variations in assessment methods.
3. Comparison within subject and instructional methods design: To ensure fairness and provide all students with a comparable learning experience, the same group of 60 students was exposed to both instructional methods, but across different thematic content. Specifically:

- The study condition involved teaching the *Painless Vision* theme using the e-Learning by Concordance (e-LbC) approach, with performance assessed through a Script Concordance Test (SCT).
- The control condition involved teaching the *Painful Vision Loss* theme using an interactive lecture, also assessed using the same SCT format.

This within-subjects design allowed for a direct comparison of instructional methods while ensuring consistency in participant exposure and assessment.

The students were selected based on the following inclusion and exclusion criteria: students who were enrolled in the Ophthalmology clerkship during the academic year 2021–2022, students who consented to participate in the study, students who completed both the pre-test and post-test assessments, and students with no prior exposure to the content of the study in previous academic years. Exclusion criteria included students who were not enrolled in the Ophthalmology clerkship during the academic year 2021–2022 and students who did not consent to participate in the study.

**Fig. 1:**
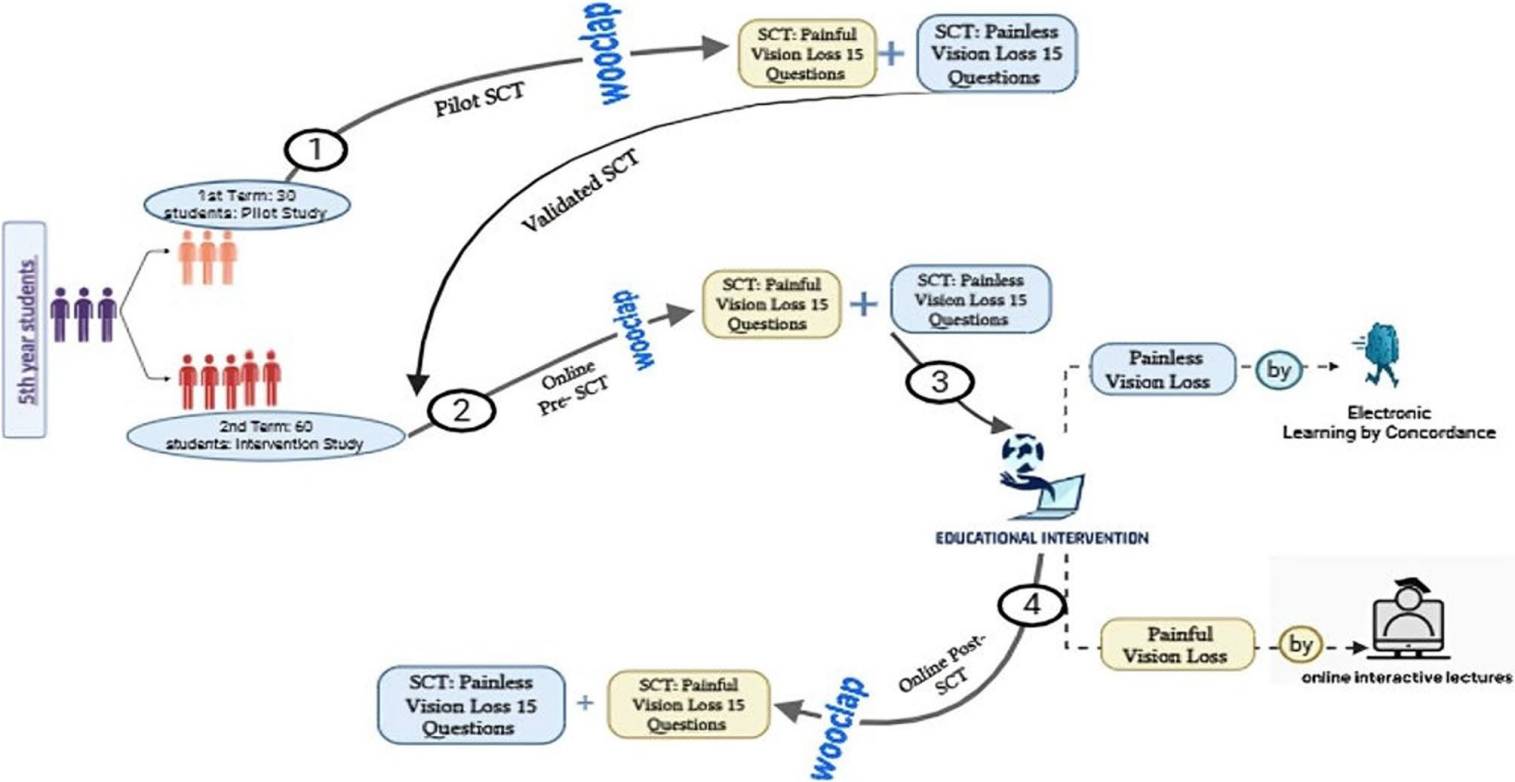
The study framework (Figure Created with BioRender.com.)

The study design was composed of three phases, Pre-intervention, intervention, and post-intervention phases, as shown in Fig. 1.

### Pre- and post-intervention phases

The student’s clinical reasoning skills were assessed using an electronic Script concordance test. The test was administrated using an online electronic platform (Wooclap) [16]. The Pre- and Post-SCT were conducted in an online proctored and closed-book setting in the faculty electronic exam hall. The students were provided with general guidelines before starting the exam. Then, they were requested to complete the SCT individually within one and half hour frame.

#### Script concordance test

Despite several tools to assess clinical reasoning skills, a series of studies have illustrated that the script concordance test (SCT) has interesting psychometric properties, in terms of reliability, face validity, and construct validity. The SCT was developed based on the illness script theory as well. It was designed to assess clinical reasoning skills & measure the degree of concordance that exists between examinees’ scripts and the panels of experts. It can be used in a paper or electronic format [21, 22].

Each test item of the SCT was designed to have four components: (1) the patient’s presentation (2) a diagnostic hypothesis, an investigation action, or a treatment option provided that is relevant to the particular situation, (3) new information is introduced in the form of a condition that might affect the diagnostic hypothesis, investigative action, or treatment option, and (4) a 5-point Likert-type scale is used to record the examines’ response. The examiner’s task was to assess the effect of the new information on the status of a diagnostic hypothesis, an investigation action, or a treatment option given.

The online ophthalmology version of the Script concordance test consists of two sections of a total of 30 vignettes and a total number of 76 items (Supplementary 1). The SCT was designed to assess diagnostic, investigative and treatment goals. The developed SCT was divided based on the major theme “Vision Loss” into two sections. The first section, containing seven clinical scenarios with fifteen vignettes of two to three items each, was developed to assess students’ clinical reasoning skills in relevance to the painful vision loss theme that was instructed by interactive lectures. The second section, containing eight clinical scenarios with fifteen vignettes of two to three items each, was developed to assess students’ clinical reasoning skills in relevance to the painless vision loss theme that was instructed by the electronic Learning by Concordance (e- LbC) approach.

#### Validation

To ensure the validity and reliability of the SCT used in this study, a multi-step validation process was conducted involving expert review and statistical analysis.

### Expert panel review for face and content validity

The SCT was initially developed based on the illness script theory and reviewed by a panel of 10 subject matter experts in ophthalmology and medical education. These experts were selected based on their extensive experience in clinical reasoning and SCT development. They assessed the test items for relevance, clarity, and alignment with real-world clinical scenarios. Each expert provided qualitative feedback on the appropriateness of the cases, the plausibility of diagnostic options, and the wording of SCT items. Necessary modifications were made based on their input to enhance clarity and ensure clinical authenticity.

### Pilot testing for construct validity

A pilot study was conducted with 30 students, separate from the study participants, to further evaluate the SCT’s validity. These students completed the SCT under exam conditions, and their responses were compared with those of the expert panel. A statistically significant difference (*p* < 0.05) between expert and student responses was observed. As script concordance is based on the assumption that examinees with more evolved illness scripts interpret data and make decisions in uncertain situations that increasingly agree with those of experienced clinicians given the same clinical scenarios, and that performance of these skills can be measured using a five-anchor Likert type scale [6]. The observation that SCT scores consistently tend to rise with increasing level of training validates this inference [23].

### Reliability testing

The internal consistency of the SCT was measured using Cronbach’s alpha, which yielded a coefficient of 0.819, indicating good reliability.

#### Scoring

We invited ten ophthalmology expert faculties to answer the SCT (Reference Panel). These experts were selected based on their extensive experience in clinical reasoning and SCT development. The aggregate method was used to develop the key score in which participants’ answers are compared to those given by a reference panel. The correct answer for an SCT was weighted based on expert response. For each answer, the credit is the number of members that chose that answer, divided by the modal value for the question [23]. With this method, all questions have the same maximum (1) and minimum (0) value. Scores obtained on each question are added to obtain a total score for the test. This number is then divided by the number of questions and multiplied by 100 to get a percentage score [24].

#### Wooclap application

Wooclap is an interactive online platform for enhancing classroom interactions and assessing students’ comprehension in real-time, through the usage of cell phones or laptops. Wooclap employs straightforward methods to make studying more engaging for students.

**Fig. 2:**
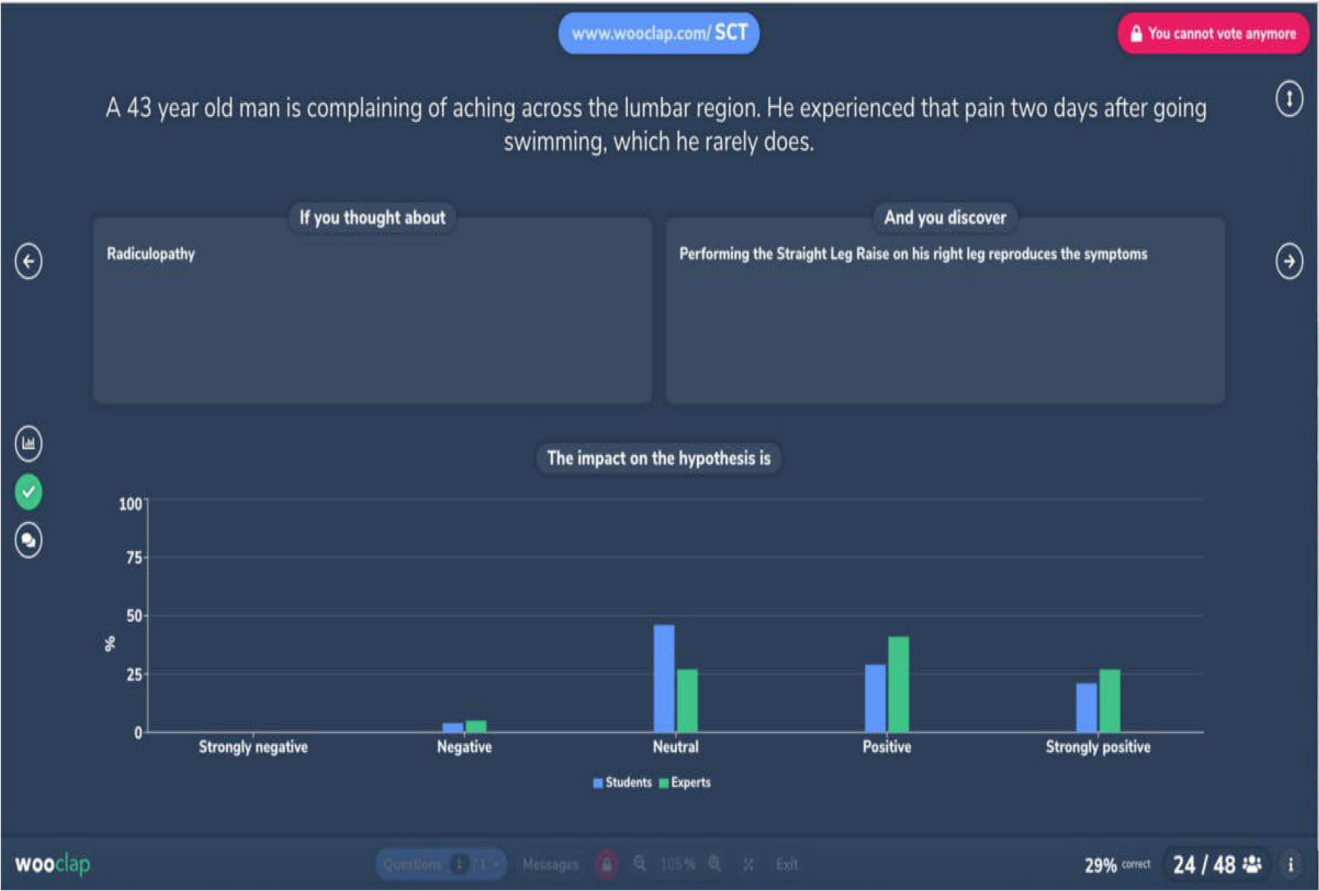
The online electronic platform (Wooclap)

It enables educators to design multiple interactive questions. Wooclap was selected due to its unique feature of having an integrated template for SCT (Script Concordance Test) within the system. This specific template was created by Bernard Charlin. It simplifies the process of adding the vignette and three columns to the test. Furthermore, the Likert scale integrated into Wooclap enables us to perform the hypothesis testing required for the exam. Students respond to these questions by mobile device, tablet, or computer. The findings are then presented live on the teacher’s presentation screen. Wooclap allows tutors to collect, display, and compare the answers of students and experts on a single platform and conduct Script concordance exams online as shown in Fig. 2.

When creating a Script Concordance Test on Wooclap, there are two possibilities: either you have already obtained the opinions of the experts through some other method, or you will collect that data using Wooclap. In the current study we have followed the first option and collected the data from the panel of experts then used Wooclap to collect the students’ answers and compare them to the experts following these steps:

1. You have already gathered the data from the panel of experts.

 Step 1: Create the question.

Select the Script Concordance Test in the list of interactions.

Then, fill in the required fields (i.e. the case description, the hypothesis and the additional information), and specify how many experts have selected each answer on the Likert scale.

 Step 2: Ask the question to your live audience.

Use the “correct answer” button to display the experts’ opinions alongside the students’.

answers [25].

We used the Wooclap platform to administrate the Script Concordance Test (SCT) electronically and synchronously at the end of each session. This tool facilitated real-time data collection by allowing both students and expert panel members to submit their responses through the application. Wooclap enabled the automatic compilation and comparison of student scores with expert reference scores, providing immediate visualization of concordance levels between learners and experts.

### Intervention phase

**Fig. 3:**
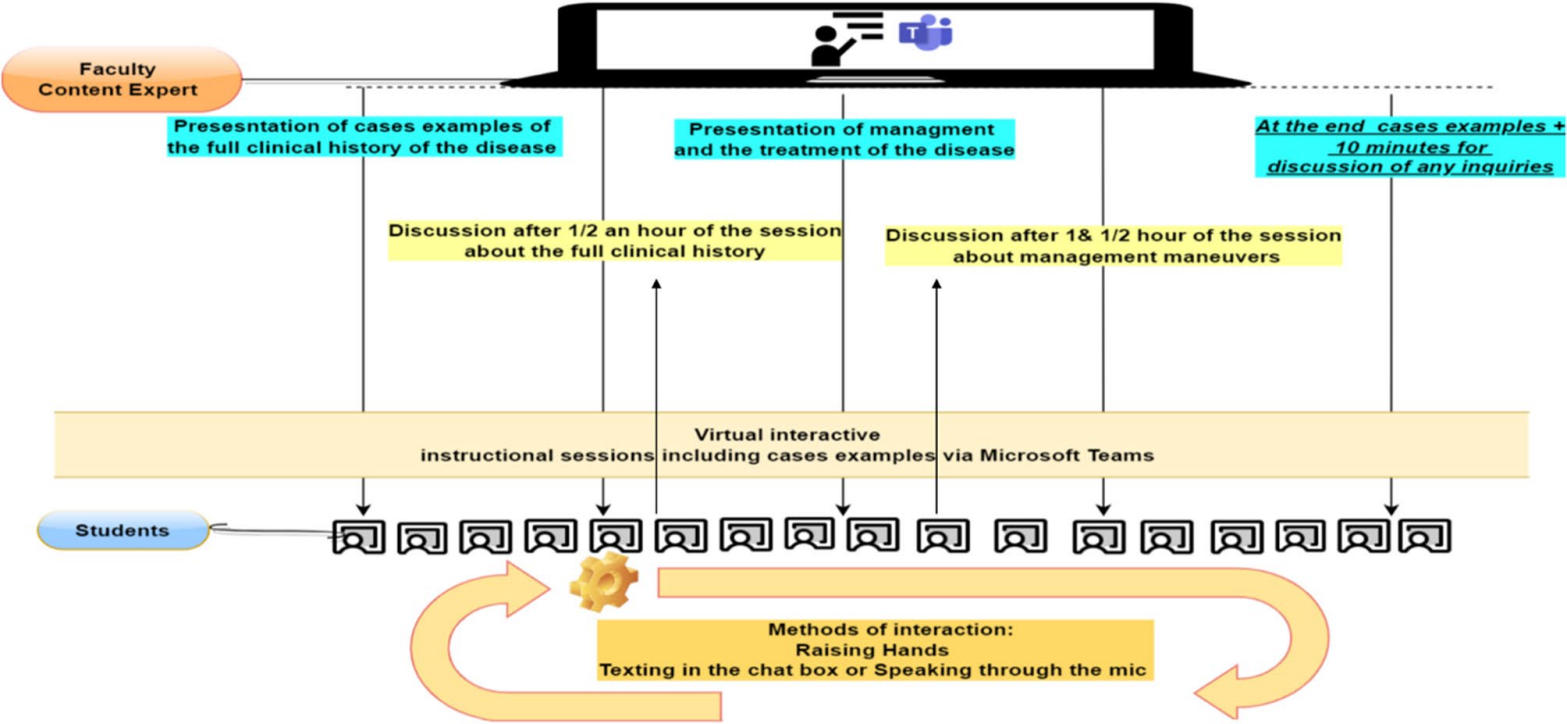
Painful vision Loss cases instructed by online interactive lectures via Microsoft Team

**Fig. 4:**
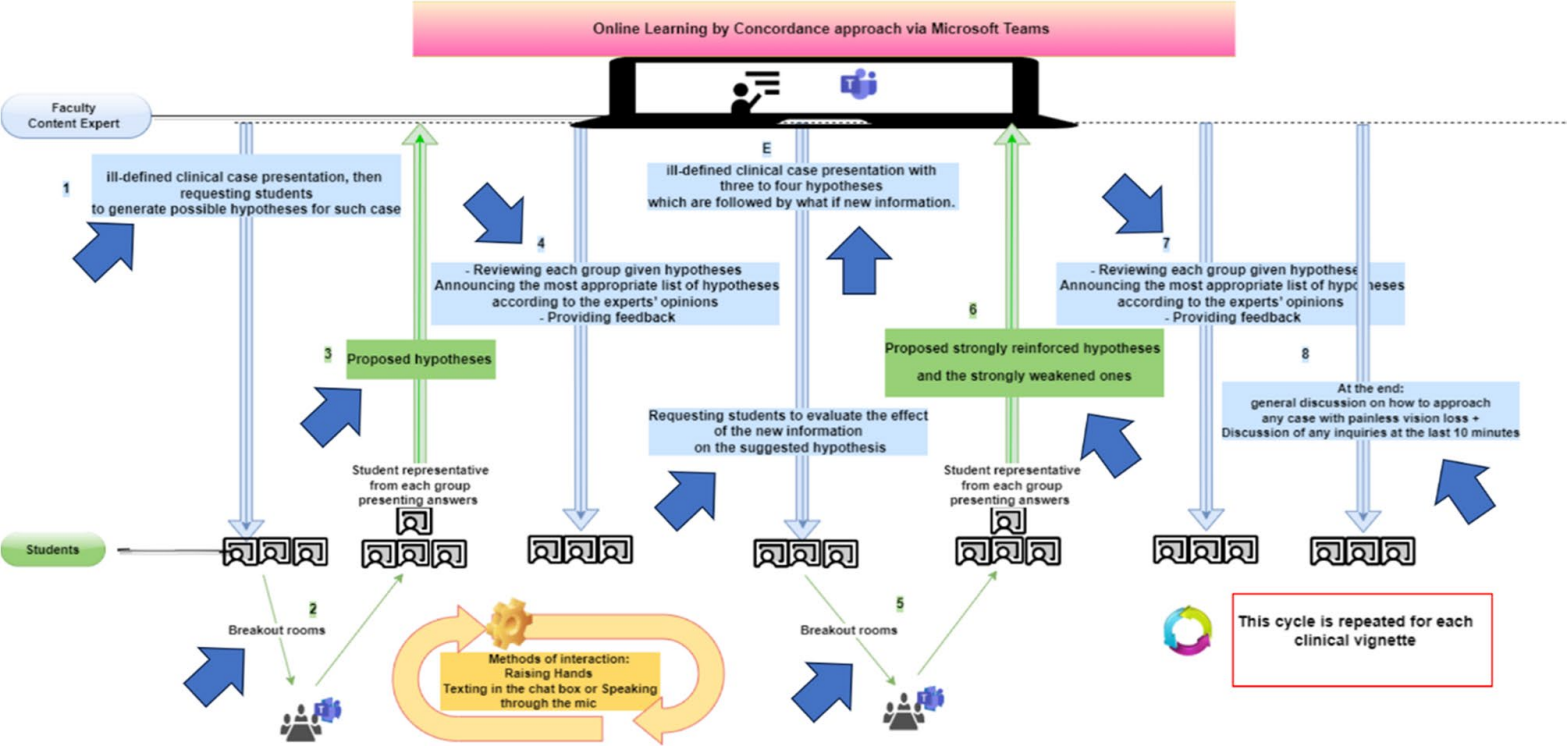
Painless vision Loss cases instructed by online LbC approach via Microsoft Teams

Following the pre-test using online SCT, the intervention phase follows. One major theme (vision loss) of ophthalmology was chosen and was divided into two sub-themes. The sub-theme, painful vision loss was instructed by online interactive lectures followed by case examples as shown in Fig. 3. The other sub-theme, painless vision loss was instructed by e-LbC as shown in Fig. 4. Both themes were demonstrated in instructional sessions and were led by a content expert in the ophthalmology field as shown in Table 1.

**Table 1:** Description of the two instructional methods used in the study: *online interactive lectures* approach for painful vision loss and e-Learning by concordance (e-LbC) approach for painless vision loss

Component	online interactive lectures (Painful Vision Loss)	e-LbC Approach (Painless Vision Loss)
Delivery Mode	Online via Microsoft Teams	Online via Microsoft Teams
Duration	2 h	2 h
Facilitation	Led by content expert and faculty instructor	Led by content expert and faculty instructor
Instructional format	Interactive lectures including case examples were the already used instructional method in the curriculum.	The e-LbC methodology integrated the principles of script concordance, allowing students to reflect on expert reasoning patterns while engaging in peer-led discussions. Feedback from both peers and content experts was integral to reinforcing learning and clarifying misconceptions. The final structure of each case included: (1) an initial ambiguous presentation, (2) a list of student-generated hypotheses, (3) new “what-if” clinical information, and (4) a reflection and justification segment.
Start	The expert gave a lecture using PowerPoint presentation about the clinical presentation of the disease of interest in 30 min, and the expert presented the management and treatment of such disease.	The first part presented to the large group included simulated clinical situations which consisted of complex or incomplete information. In the large group, the students were requested to formulate the possible diagnostic hypotheses of such an ill-defined case. To do what was requested, they were assigned to breakout rooms. The breakout room function on Microsoft Teams was used to virtually divide students into small groups of 5–7 students for each activity requested, after which they would return to the large group. The role of the faculty instructor was just monitoring the rooms without participation to see how students reached the differential diagnosis.
During the session	Following this, a 60-minute case-based discussion was held, where the content expert walked students through real clinical scenarios, prompting learners to propose diagnoses and outline diagnostic steps. there was an interactive discussion between the expert and the students on the manoeuvre of each examination lab test or imaging that would be requested for the disease	In breakout rooms, Students read the case and use online resources (such as search engines) to provide diagnostic hypotheses of such ill-defined cases. Back in the large group, student representatives from each room discussed the possibilities and proposed a list of diagnostic hypotheses to justify their choices. At the end of this section, the content expert reviewed each diagnostic hypothesis given, provided feedback, and omitted incorrect answers. In the second section of e-LbC, the expert started by presenting the collected list of the diagnostic hypotheses for such ill-defined cases in the form of 4 or 6 diagnostic hypotheses. Then, these 4 or 6 hypotheses were followed by new “what if " information. Following that, the third section of e-LbC is the judgment expected of the students. They were requested to evaluate the effect of the new information on the suggested hypotheses. Again, in the breakout rooms, they tried to evaluate the effect of the new information on the suggested hypotheses. They excluded some of the proposed hypotheses as strongly weakened. While they included some hypotheses as strongly reinforced. Back in the large group, student representatives from each room presented the proposed strongly reinforced hypotheses and the strongly weakened ones based on their evaluation of the effect of the new information on the list of suggested hypotheses.
At the end of the session	The experts presented the treatment of such a disease and gave the students 10 min to ask any questions about any part they hadn’t understood. This structure ensured a high level of fidelity in implementing the lecture-based method with consistent opportunities for student engagement and expert feedback.	At the end of this section, the content expert reviewed each group’s given answers announced the most appropriate answers according to the experts’ opinions and provided them with feedback from those experts. The above sections were repeated for investigative and treatment purposes. All the responses were discussed with the students and the expert. The expert was keen on probing the rationale of each student for the selection of one answer. At the end of the whole session, the content expert reviewed the whole session. He summarized the session by how to approach any case with painless vision loss, listing the causes of painless loss of vision, what are the steps in the workup and what is the list of possible treatments according to each cause. Then, the expert gave the students 10 min to ask any questions about any part they hadn’t understood.
Interactivity	Live polls, Q&A	Peer discussion in breakout rooms, expert feedback, critical judgment tasks
Case Focus	Conditions with **painful vision loss**	Conditions with **painless vision loss**
Case Examples	Acute Angle-Closure Glaucoma, Anterior Uveitis (Iritis), Endophthalmitis, Scleritis	Central retinal vein occlusion, retinal detachment, optic neuritis, ischemic ON
Assessment Method	Q&A and interaction during session	Script concordance reasoning steps + peer & expert feedback
Final Summary	10-minute Q&A and summary	Expert-led case wrap-up, full case review, Q&A
Engagement Strategy	Structured Q&A, prompting less vocal students	Reflection, justification, expert probing of reasoning
Feedback Approach	Real-time feedback from expert	Peer and expert feedback on reasoning at each stage

### Evaluation of the student perceptions

At the end of the post-SCT, students’ perception towards the electronic LbC was assessed by using an anonymous questionnaire. The questionnaire was developed by the authors to explore the student perception. The questionnaire was composed of 24 items/questions and was adopted from previous studies [16, 17]. To ensure face and content validity, the questionnaire underwent a validation process involving ten medical education experts with significant experience in SCT. The experts received the questionnaire and completed an evaluation via an online form, which included an area for suggestions for improvement. Their feedback was used to refine and finalize the questionnaire, ensuring its relevance and clarity. The final version of the validated questionnaire was then administered to the students. At the end of the session, students were reminded to complete the perception questionnaire, as outlined and approved in the informed consent. The questionnaire was administered via Microsoft Google Forms disseminated through a shared link once, at the end of the sessions. All the students in the study group were informed about the questionnaire and received it. 60 students respond with response rate 100%. No missing data were identified, so all the completed questionnaires were included in the analysis. Students assessed each statement using a 5-point Likert-type scale from strongly disagree (1) to strongly agree (5).

### Statistical analysis

Data were analysed with descriptive and analytic statistics such as paired t-tests using SPSS, version 24. P values less than 0.05 were considered significant. The effect size was measured by Cohen’s d method.

## Results

The study compared two different educational methods across two themes. Both were evaluated against each other before and after learning using the script concordance test (SCT). The results were discussed in five sections, covering changes in overall SCT scores, pre- and post-SCT scores, comparisons within and between groups, vignette analysis, and students’ perceptions.

### Overall SCT score improvement (Table 2)

Given that the total SCT scores were normally distributed, parametric tests were applied for their comparison. Students in both groups demonstrated statistically significant improvements in post-SCT scores relative to their pre-SCT scores (Table 2). Moreover, the e-LbC, painless vision loss theme, achieved a greater improvement, with a large effect size (Cohen’s d = 4.16), compared with the interactive lecture, painful vision loss theme, (Cohen’s d = 3.75).

### Within-group comparisons (Table 2)

The average change in SCT scores was 17.45 (95% CI: 16.39–18.51) in the e-LbC group and 12.37 (95% CI: 11.54–13.20) in the lecture group, yielding a Cohen’s d of 1.30, which reflects a large effect size, reinforcing the instructional advantage of e-LbC (Table 2).

**Table 2:** Comparison between the total scores of the Pre-test and post-test script concordance test in each group by pair T-test and between groups by students T-test

Groups	Number		Mean (SD)	95% Confidence Interval		*p*-value	Effect size (Cohen’s D)
Painless vision loss theme ^1,2^	60					< 0.001***	**4.16**
Pretest			7.73 (1.18)	7.43–8.03			
Posttest			25.18 (3.76)	24.23–26.13			
Painful vision loss theme ^1,2^	60					< 0.001***	**3.75**
Pretest			7.83 (1.24)	7.52–8.14			
Posttest			20.19 (2.83)	19.47–20.91			
Pretest ^,4^	60					**0.667**	**− 0.08**
Painless vision loss theme			7.73 (1.18)	7.43–8.03			
Painful vision loss theme			7.83 (1.24)	7.52–8.14			
Posttest ^3,4^						< 0.001***	**1.50**
Painless vision loss theme			25.18 (3.76)	24.23–26.13			
Painful vision loss theme			20.19 (2.83)	19.47–20.91			
Change in Total SCT Scores	60					< 0.001***	**1.30**
Painless vision loss theme			17.45 (4.19)	16.39–18.51			
Painful vision loss theme			12.37 (3.29)	11.54–13.20			

Interpretations of Cohen’s d:A value of 0.2: represents a small effect size.A value of 0.5: represents a medium effect size.A value of 0.8: represents a large effect size

Parametric data

Paired t-test ^1^, P value statistically significant (*P* ≤ 0.001)***

Note.^2^ Hₐ: Post Scores > Pre-Scores

Independent t-test ^3^, P value statistically significant (*P* ≤ 0.001)***

Note.^4^ Hₐ: Painless vision loss > Painful vision loss

Note.^5^ Hₐ: Experts’ SCT > Students’ SCT Scores

### Between-group comparison (Fig. 5; Table 2)

**Fig. 5:**
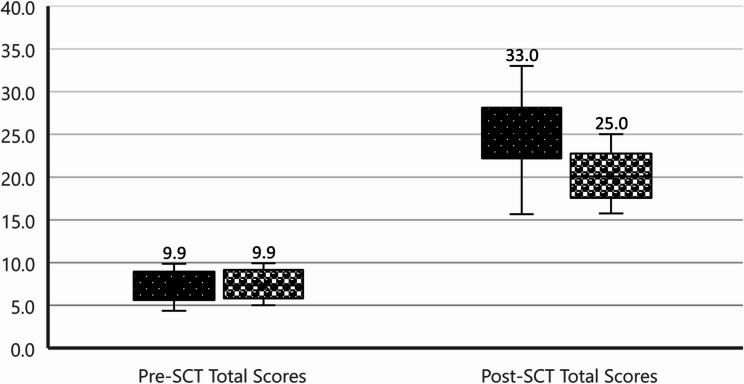
Differences in the students’ total scores of Pre-SCT and Post-SCT between painless and painful groups

While baseline (pre-SCT) scores were comparable between the two groups (*p* = 0.667), the post-SCT scores were significantly higher in the painless vision loss theme (mean = 25.18) compared to the painful vision loss theme (mean = 20.19), indicating superior performance following e-LbC instruction (Fig. 5; Table 2). This analysis assumed normality of the data, which was verified using the Shapiro-Wilk test. The Shapiro-Wilk test indicated that the data were normally distributed (*p* = 0.03) supporting the assumption of normality required for the paired t-test.

### Item-level (Vignette) analysis (Table 3)

Unlike the total SCT scores, which were normally distributed and therefore suitable for parametric tests, the individual vignette scores did not meet normality assumptions. The requirements for the Mann–Whitney U test, which does not assume normality, were satisfied, as the Shapiro–Wilk test (*p* = 0.56) confirmed that the 30 vignette scores of the developed SCT were non-normal distributed. Accordingly, post-SCT analysis showed that students in the e-LbC group outperformed their peers in 10 of the 15 vignettes, with statistically significant differences in 8 (*p* < 0.05), highlighting e-LbC’s effectiveness across diverse clinical contexts (Table 3).

**Table 3:** Differences between vignettes’ scores of painless and painful vision loss themes in the post-SCT

Vignette	Median (IQR)		*p* value
	Post-SCT scores in painless vision loss theme	Post-SCT scores in painful vision loss theme	Painless theme vs. Painful theme in post-SCT ^3,4^
1	1.25 (1.00)	0.68 (0.82)	0.999
2	1.67 (0.84)	1.00 (1.00)	0.377
3	1.54 (1.00)	0.43 (1.10)	< 0.001***
4	2.10 (1.03)	0.67 (1.10)	< 0.001***
5	2.00 (1.24)	0.25 (1.00)	< 0.001***
6	2.00 (1.02)	0.00 (0.43)	< 0.001***
7	1.43 (0.75)	0.00 (0.43)	< 0.001***
8	2.11 (1.21)	0.43 (0.92)	< 0.001***
9	1.34 (1.00)	0.67 (1.00)	0.972
10	2.25 (1.65)	0.25 (0.50)	0.006
11	1.43 (1.00)	0.00 (0.25)	0.766
12	1.68 (1.81)	0.50 (0.75)	0.391
13	1.67 (1.00)	0.25 (0.50)	0.378
14	2.25 (1.33)	0.25 (0.50)	0.115
15	2.00 (0.90)	0.00 (0.54)	0.007

**Non-Parametric data**

Mann–Whitney U test ^3^, P value statistically significant (*P* ≤ 0.001). ***

Note. ^4^ Hₐ: Painless vision loss theme > Painful vision loss theme

### Student perception (Table 4)

The questionnaire, as shown in Table 4, results indicated the intervention of using electronic learning by concordance as an instructional method in a painless vision loss theme was generally well-received by students. The reliability coefficient of the perception questionnaire was 0.823 with good internal consistency. Most students (90%) strongly agreed or agreed that the e-LbC approach aids in improving clinical reasoning ability for the future and (62%) of students reported that they were overall satisfied with the instructional session. Moreover, most students (69%) strongly agreed or agreed that SCT can be used as a useful instructional method for the future and (75%) strongly agreed or agreed that SCT is an effective assessment tool.

**Table 4:** Students’ perception towards script concordance test and learning by concordance strategy instructional session

Items	Agreement (%)	Neutrality (%)	Disagreement (%)
Online Script Concordance Test Structure and Format:			
1. Clear test Format	80%	13%	7%
2. Clear test instruction	52%	43%	5%
3. Appropriate test duration	74%	26%	0%
4. Challenging test format	34%	25%	41%
5. Familiar clinical scenarios seen in clinical training	100%	0%	0%
6. Real-life situations reflection.	84%	16%	0%
7. Leading to learning enhancement	52%	18%	30%
8. Assessment tool for the future	75%	16%	8%
9. Useful Instructional method for the future	69%	18%	13%
10. Effective Assessment tool	66%	28%	7%
Electronic Learning by concordance strategy instructional session:			
11. Aids in gaining a good understanding of concepts in the clinical reasoning field	79%	21%	0%
12. Aids in improving clinical reasoning ability for the future	90%	5%	5%
13. Clearly defined objectives of the session	66%	23%	12%
14. Reasonable amount of material delivered in the session	95%	5%	0%
15. Appropriate level of difficulty of the session	92%	8%	0%
16. Good Learning Experience	44%	28%	28%
17. Aids in improving current clinical reasoning skills	74%	16%	10%
18. Leading to learning enhancement	56%	34%	10%
19. Appropriate feedback was given for the answers during the session	41%	36%	23%
20. Boring and wasting of time than other instructional methods	8%	31%	61%
21. Encouraging student participation	51%	23%	26%
22. Recommended type of instructional method for illustration of other signs and symptoms	95%	5%	0%
23. Overall, Satisfaction towards this learning strategy	62%	38%	0%

## Discussion

This study introduces a novel electronic approach to teaching clinical reasoning in ophthalmology using the Learning by Concordance (e-LbC) method. It promotes student-centered, active engagement by allowing learners to apply knowledge in real time and receive immediate expert feedback through the Wooclap platform. This feature was especially beneficial during the remote learning challenges posed by the COVID-19 pandemic.

While this study focused on ophthalmology, the structured design of e-LbC, rooted in illness script theory and dual-process reasoning, makes it broadly applicable across medical specialties that involve diagnostic uncertainty or complex clinical judgment. Its adaptability across educational levels allows tailored implementation: novice learners can engage with guided cases and detailed feedback, while advanced learners can benefit from complex, ambiguous cases that support the transition from intuitive (Type 1) to analytical (Type 2) reasoning [3]. This adaptability was also observed in studies by Lafond et al. [26] and Charton et al. [10], who successfully implemented e-LbC in pulmonary and ECG training, respectively. Similarly, Vaillant-Corroy et al. [27] demonstrated that LbC can effectively foster the development of professionalism in dentistry, highlighting its versatility across different health professions and educational contexts.

Our findings highlight the potential of e-LbC to strengthen clinical reasoning, as shown by the significant improvement in post-SCT scores, particularly in the painless vision loss theme instructed using e-LbC. This improvement may be attributed to the contextual, dynamic learning environment that mirrors real-life scenarios and supports illness script formation [28]. A systematic review also emphasized the value of structured reasoning programs in facilitating the transition from pre-clinical to clinical learning [29]. A Cohen’s d of 4.16, as observed in the e-LbC painless vision loss theme, is considered an exceptionally large effect size, far exceeding the conventional threshold of 0.8 for a large effect. This suggests that the improvement in students’ clinical reasoning skills following the e-LbC intervention was not only statistically significant but also educationally and practically meaningful. Such a large effect reflects an improvement in performance, indicating that the instructional method influenced students’ ability to reason through clinical cases involving diagnostic uncertainty [30]. This discrepancy may also reflect a novelty effect—when learners encounter a new, interactive instructional method, their initial engagement and performance can be unusually high, even if the effect may diminish over time [31].

Furthermore, the confidence intervals for the change in SCT scores were narrow and non-overlapping (e-LbC: 95% CI 16.39–18.51; lecture: 95% CI 11.54–13.20), demonstrating a high level of precision in the estimates and implying that the observed differences between groups are statistically significant. The consistency of these intervals adds to the strength of the findings, as even the most cautious estimations show a significant improvement, particularly in the e-LbC group. This strengthens the intervention’s practical instructional impact.

Students appreciated the ability to compare their reasoning to that of expert panels, which reinforced their metacognitive skills and supported ongoing development through practice and feedback. This is consistent with Fernandez et al. [7], who noted that LbC fosters reflective thinking. Moreover, as Deschênes et al. [11] and Lecours et al. [5] reported, online LbC tools in clinical domains like dermatology helped students monitor their understanding and refine clinical knowledge through expert justifications.

Importantly, the e-LbC method also supports the development of learners’ tolerance for clinical uncertainty—a crucial competency in medical education. Encouraging the expression of uncertainty during LbC activities allows students to normalize and address it, as supported by research linking intolerance of uncertainty with poor clinical decision-making, burnout, and cognitive errors [30, 32]. However, the effectiveness of e-LbC may be influenced by factors such as prior knowledge, cognitive load, and case complexity, indicating the need for further research to optimize implementation conditions. Designing Learning-by-Concordance (LbC) cases presents the challenge of creating clinical scenarios that guide students in developing reasoning for complex and uncertain situations. LbC is particularly suited for cases where multiple responses may be appropriate, emphasizing the reasoning process over identifying a single correct answer [9].

The inclusion of SCTs, administered electronically via Wooclap, not only provided a methodologically aligned assessment strategy but also enhanced learner engagement. Students valued the SCT format both as a learning and assessment tool, aligning with findings by Lineberry et al. [33], and highlighting SCT’s role in promoting deeper understanding and reflective learning.

Overall, this study supports the scalability and practicality of e-LbC as an instructional strategy. Its simplicity, cost-effectiveness, and capacity to accommodate various group sizes make it suitable for diverse medical education settings. Student feedback in this study strongly endorsed the e-LbC method, consistent with findings across specialties that highlight its usability and pedagogical value [12, 33, 34].

### Limitations

This study has some limitations, including a small, single-cohort sample within the ophthalmology specialty, which limits the generalizability of the findings. The quasi-experimental design without randomization may have introduced selection bias and using different forms for pre- and post-SCT questions raises concerns about test equivalence. Additionally, since item-level analyses involved multiple comparisons, the risk of Type I errors cannot be ruled out, so the results should be interpreted with caution. Relying on self-reported, non-validated feedback also introduces response bias. The study may have emphasized positive outcomes while overlooking other explanations. Moreover, the lack of long-term follow-up limits insights into knowledge retention. While dividing the SCT into “painless” and “painful” versions helped differentiate themes, it might have introduced content-related bias. However, both versions were carefully matched in structure and difficulty, and baseline scores indicated similar initial knowledge levels. The significant improvement in the painless vision loss theme suggests the instructional method influenced the results. Another limitation is potential crossover effects, since the same students experienced both instructional methods (e-LbC and interactive lectures) on different topics. Although thematic separation was maintained, some overlap in knowledge or reasoning strategies may have occurred.

Additional strengths in this study included that our study is the first to implement in Ophthalmology, has strong study design, introduced students to two different teaching approaches, and used a valid and reliable assessment method. Moreover, use of Wooclap helped in recording and comparing the scores. However, future studies should include randomized designs, larger and more diverse samples, validated assessment tools, and incorporate qualitative and long-term follow-up analyses.

## Conclusion

This study highlights the promise of combining the e-LbC method with SCT as a novel and effective way to develop clinical reasoning in Ophthalmology. Students valued both approaches pedagogically and suggested using them for other clinical subjects in their curriculum. As medical education advances, especially amid challenges like the COVID-19 pandemic, electronic formats such as e-LbC offer affordable and flexible solutions suitable for both small and large groups of students.

## Supplementary Information

Supplementary Material 1.

Supplementary Material 2.

## Data Availability

Data can be obtained from the corresponding author upon request.

## Abbreviations

SCT: Script Concordance Test
e-LbC: Electronic Learning by Concordance
CR: Clinical Reasoning
