# Supplementary material for: A digital recipe for enhancing clinical reasoning: the role of e-learning by concordance (E-LbC): a quasi-experimental study
Source: BMC Med Educ. 2025 Oct 2;25:1279. doi: 10.1186/s12909-025-08005-w (PMC12490060; doi:10.1186/s12909-025-08005-w)
Supplement: Supplementary file 1 — Supplementary Material 1. [file 12909_2025_8005_MOESM1_ESM.docx]

**Students’ Satisfaction survey/questionnaire:**All medical students in both groups will complete a satisfaction questionnaire/survey about the illness script approach and SCT approach. Students assessed each statement using a 5-point Likert-type scale from strongly disagree (1) to strongly agree (5).

| **Statements** | **Response** | | | | |
| --- | --- | --- | --- | --- | --- |
|  | Strongly Disagree | Disagree | Neutral | Agree | Strongly Agree |
| **Students’ satisfaction with SCT Exam.** |  |  |  |  |  |
| 1. The test format is clear |  |  |  |  |  |
| 1. The test instructions are clear |  |  |  |  |  |
| 1. The duration of the test is appropriate |  |  |  |  |  |
| 1. The test is challenging to be answered |  |  |  |  |  |
| 1. The test includes a familiar clinical scenario that you have seen during your training |  |  |  |  |  |
| 1. The test reflects ‘real-life’ situations |  |  |  |  |  |
| 1. I think this method would enhance my learning |  |  |  |  |  |
| 1. I prefer the use of this test as an assessment method |  |  |  |  |  |
| 1. I prefer the use of this test as a teaching method |  |  |  |  |  |
| 1. I find it effective and useful to be assessed by this method in the future |  |  |  |  |  |
| **Students’ satisfaction with the learning by concordance approach as an instructional method.** |  |  |  |  |  |
| 1. I gained a good understanding of concepts in the clinical reasoning field. |  |  |  |  |  |
| 1. The Learning by concordance approach as an instructional method encouraged me to improve my clinical reasoning ability in the future. |  |  |  |  |  |
| 1. The learning objectives of sessions using Learning by concordance approach as an instructional method were clearly defined. |  |  |  |  |  |
| 1. The amount of material delivered in the sessions was reasonable |  |  |  |  |  |
| 1. The level of difficulty of the sessions was appropriate. |  |  |  |  |  |
| 1. The sessions using Learning by concordance approach as an instructional method were a good learning experience for me. |  |  |  |  |  |
| 1. The learning by concordance approach as an instructional method was useful for improving my clinical reasoning skills |  |  |  |  |  |
| 1. This type of instructional method emphasized learning |  |  |  |  |  |
| 1. The tutor gave us appropriate feedback about the answers |  |  |  |  |  |
| 1. The session was boring and wasted my time |  |  |  |  |  |
| 1. The students’ participation was encouraged |  |  |  |  |  |
| 1. I recommend this type of instructional method for illustration of other signs and symptoms. |  |  |  |  |  |
| 1. Overall, I am satisfied with the Learning by concordance approach as an instructional method. |  |  |  |  |  |
