# Supplementary material for: A digital recipe for enhancing clinical reasoning: the role of e-learning by concordance (E-LbC): a quasi-experimental study
Source: BMC Med Educ. 2025 Oct 2;25:1279. doi: 10.1186/s12909-025-08005-w (PMC12490060; doi:10.1186/s12909-025-08005-w)
Supplement: Supplementary file 2 — Supplementary Material 2. [file 12909_2025_8005_MOESM2_ESM.docx]

**Script concordance test for undergraduate students**

**Test theme: Painful vision loss**

Name: Date:

The test contains seven clinical scenarios with fifteen vignettes of two to three items each. The vignettes are about the **diagnosis** of common problems, **investigations,** and **treatment**.

After reading the scenario of each item, please highlight the degree of agreement or disagreement with the hypothesis (the second column contains information that helps you to include or exclude the corresponding differential diagnosis).

Please make your choice of the most adequate answer. The hypothesis will become:

**-1 = unlikely (ruled out or almost ruled out)**

**0 = neither likely nor unlikely**

**+1 = likely**

| **Patient (1)** | | | | | | | |
| --- | --- | --- | --- | --- | --- | --- | --- |
| A squash player presented with defective vision after the squash ball hit his right side of his face. | | | | | | | |
| **If patient complained of** | **And by examination, you found** | | **This hypothesis would become** | | | | |
| Defective vision | Hyphema | | | -1 | 0 | | +1 |
| Headache and ocular pain | Angle recession | | | -1 | 0 | | +1 |
| Photophobia | Mydriasis | | | -1 | 0 | | +1 |
| **If you requested** | **And then you found** | **This hypothesis would become** | | | | | |
| Ocular ultrasound | Vitreous hemorrhage | | | -1 | | 0 | +1 |
| CT orbit | Inferior orbital floor fracture | | | -1 | | 0 | +1 |
| After hospital admission and patient management, he was discharged with 6/6 vision. However, he returned after 3 moths complaining of diminution of vision in the same eye | | | | | | | |
| **If you are thinking of** | **And then you found** | | **This hypothesis would become** | | | | |
| Complicated cataract | The patient complained of Foggy vision | | | -1 | | 0 | +1 |
| Secondary glaucoma | Opacified crystalline lens | | | -1 | | 0 | +1 |

| **Patient (2)** | | | | |
| --- | --- | --- | --- | --- |
| Forty years old farmer presented to the emergency department after having blunt trauma to his right eye by a piece of wood. On examination, you noticed corneal epithelial defect and stromal infiltrate. | | | | |
| **If you were thinking of** | **And if the patient had** | **This hypothesis would become** | | |
| Dendritic corneal ulcer | Satellite lesions | -1 | 0 | +1 |
| Fungal keratitis | Discomfort or minimal pain | -1 | 0 | +1 |
| Bacterial keratitis | Hypopyon in the anterior chamber | -1 | 0 | +1 |
| **Then you considered treating this patient** | | | | |
| **If you were thinking of** | **And the patient is treated with** | **This hypothesis would become** | | |
| Dendritic corneal ulcer | Systemic and topical natamycin | -1 | 0 | +1 |
| Fungal keratitis | Systemic and topical amphotericin B | -1 | 0 | +1 |

| **Patient (4)** | | | | | | |
| --- | --- | --- | --- | --- | --- | --- |
| A fifty-year-old diabetic woman presented with bilateral ciliary injection, muddy iris, keratic precipitates and painful vision loss. | | | | | | |
| **If you were thinking of** | **And then you found** | **This hypothesis would become** | | | | |
| Secondary glaucoma | Posterior psynechia | -1 | 0 | | | +1 |
| Endophthalmitis | Clear vitreous on ultrasonography | -1 | 0 | | | +1 |
| Phacoanaphylaxis | No history of trauma | -1 | 0 | | | +1 |
| **If you were considering treatment with** | **As it had a significant effect in** | **This hypothesis would become** | | | | |
| Topical atropine | Reducing pain | -1 | | 0 | +1 | |
| Topical steroids | Reducing formation of psynechia | -1 | | 0 | +1 | |
| Topical pilocarpine | Reducing intraocular pressure | -1 | | 0 | +1 | |

| **Patient (5)** | | | | |
| --- | --- | --- | --- | --- |
| A 44-year-old lady presented to the emergency department with severe left ocular pain of 2 hours duration and 1 attack of vomiting. On examination, she had severely elevated intraocular pressure (digitally) and corneal epithelial edema. | | | | |
| **If you were thinking of** | **And then you found** | **This hypothesis would become** | | |
| Primary angle closure glaucoma | Small axial length and hyperopia | -1 | 0 | +1 |
| Secondary angle closure glaucoma | Swollen opaque crystalline lens | -1 | 0 | +1 |
| Secondary open angle glaucoma | Traumatic mydriasis and RBCS in the anterior chamber | -1 | 0 | +1 |
| **If you were considering treatment with** | **And then you found the patient had** | **This hypothesis would become** | | |
| Mannitol 20% | Ischemic heart disease and hypertension | -1 | 0 | +1 |
| Peripheral laser iridotomy | Closed angle on gonioscopy | -1 | 0 | +1 |

**Good luck**

**Script concordance test for undergraduate students**

**Test theme: Painless vision loss**

Name: Date:

The test contains eight clinical scenarios with fifteen vignettes of two to three items each. The vignettes are about the **diagnosis** of common problems, **investigations,** and **treatment**.

After reading the scenario of each item, please highlight the degree of agreement or disagreement with the hypothesis (the second column contains information that helps you to include or exclude the corresponding differential diagnosis).

Please make your choice of the most adequate answer. The hypothesis will become:

**-1 = unlikely (ruled out or almost ruled out)**

**0 = neither likely nor unlikely**

**+1 = likely**

| **Patient (1)** | | | | |
| --- | --- | --- | --- | --- |
| A 55-year-old female with uncontrolled hypertensive patient came to the ophthalmology outpatient clinic complaining of right defective vision for three days. On examination, there was right relative afferent pupillary defect | | | | |
| **If you were thinking of** | **And then you found** | **This hypothesis would become** | | |
| Doing fundus fluorescein angiography | Hard exudates | -1 | 0 | +1 |
| Doing fundus fluorescein angiography | Flame-shaped hemorrhages and cotton wool spots | -1 | 0 | +1 |
| **After diagnosis was confirmed for central retinal vein occlusion, you must decide the suitable treatment.** | | | | |
| **If you were thinking of** | **And then you decided to** | **This hypothesis would become** | | |
| The first line of treatment | Control the systemic condition | -1 | 0 | +1 |
| Treatment of macular edema | Observation only | -1 | 0 | +1 |

| **Patient (2)** | | | | | |
| --- | --- | --- | --- | --- | --- |
| 20 years old female patient came complaining of defective vision. After complete examination, her final refraction was (-2.00) diopters for her right eye and (-18.00) diopters for her left eye. | | | | | |
| **If you were considering the management by** | **And if the patient came in her first revision feeling** | | **This hypothesis would become** | | |
| Glasses | annoyed | -1 | | 0 | +1 |
| Contact lenses | Satisfied with her vision | -1 | | 0 | +1 |
| Intraocular lens for left eye | Of defective reading ability | -1 | | 0 | +1 |

| **Patient (3)** | | | | | | |
| --- | --- | --- | --- | --- | --- | --- |
| An atherosclerotic hypertensive and diabetic patient presented with left painless drop of vision. Examination of right eye was unremarkable | | | | | | |
| **If your diagnosis were to be** | **And by examination, the findings included** | | **This hypothesis would become** | | | |
| Central retinal vein occlusion | Left relative afferent pupillary defect | | -1 | | 0 | +1 |
| Central retinal artery occlusion | Cherry red spot | | -1 | | 0 | +1 |
| Anterior ischemic optic neuropathy | Inferior visual field defect on confrontation test | | -1 | | 0 | +1 |
| **After completion of patient examination, you requested the suitable investigations.** | | | | | | |
| **If you requested** | | **And then you found** | | **This hypothesis would become** | | |
| Fundus fluorescein angiography | | Cystoid macular edema | | -1 | 0 | +1 |
| Lipid profile | | High levels of high-density lipoproteins | | -1 | 0 | +1 |
| Glycosylated hemoglobin (HbA1c) | | 8.5 % | | -1 | 0 | +1 |

| **Patient (8)** | | | | | |
| --- | --- | --- | --- | --- | --- |
| A 33-year-old obese female presented with transient obscuration of vision that lasted for few seconds. | | | | | |
| **If you were thinking of** | | **And by examination, you found** | **This hypothesis would become** | | |
| Papilledema | | Enlarged blind spot by confrontation test | -1 | 0 | +1 |
| Papillitis | | Visual acuity of 6/9 for both eyes | -1 | 0 | +1 |
| Retrobulbar neuritis | | Intact optic nerve functions | -1 | 0 | +1 |
| **After completion of patient examination, you requested the suitable investigations.** | | | | | |
| **If you requested** | **And then you found** | | **This hypothesis would become** | | |
| CT brain | Partially empty sella turcica | | -1 | 0 | +1 |
| Automated perimetry | Enlarged blind spot for both fields | | -1 | 0 | +1 |

**Good luck**
